# Intakes of Vitamin B6 and Dietary Fiber and Clinical Course of Systemic Lupus Erythematosus: A Prospective Study of Japanese Female Patients

**DOI:** 10.2188/jea.JE20100157

**Published:** 2011-07-05

**Authors:** Yuko Minami, Yasuhiko Hirabayashi, Chisato Nagata, Tomonori Ishii, Hideo Harigae, Takeshi Sasaki

**Affiliations:** 1 Division of Community Health, Tohoku University Graduate School of Medicine, Sendai, Japan; 2 Department of Hematology & Rheumatology, Tohoku University Graduate School of Medicine, Sendai, Japan; 3 Department of Epidemiology & Preventive Medicine, Gifu University Graduate School of Medicine, Gifu, Japan

**Keywords:** systemic lupus erythematosus, disease activity, atherosclerosis, vitamin B6, dietary fiber

## Abstract

**Background:**

Intakes of selected vitamins and dietary fiber may influence the clinical course of systemic lupus erythematosus (SLE). Using a cohort study method, we investigated the associations of dietary intake of vitamin B6 and B12, folate, and dietary fiber with the risk of active disease and atherosclerotic vascular events in SLE.

**Methods:**

The study included female SLE patients in the Miyagi Lupus Cohort, which was founded in 1995. Dietary nutrients at baseline were estimated by a semiquantitative food frequency questionnaire. The association of each nutrient intake with the risk of active disease was investigated in 216 patients who had inactive disease at baseline. The association with atherosclerotic vascular events was assessed in 196 women who had inactive disease and no history of atherosclerotic diseases at baseline.

**Results:**

Forty-three cases of active disease were identified during 9966 person-months of follow-up (1995–1999). During 19 575 person-months of follow-up (1995–2005), 20 atherosclerotic vascular events were documented. The Cox proportional hazards model revealed an inverse association between vitamin B6 intake and the risk of active disease (hazard ratio for the highest as compared with the lowest tertile, 0.41; 95% confidence interval, 0.18–0.97; *P* for trend = 0.04). An inverse association was also found for dietary fiber intake (*P* for trend = 0.01). However, no significant association was observed between intakes of these nutrients and the risk of atherosclerotic vascular events.

**Conclusions:**

Higher intake of vitamin B6 and dietary fiber may prevent the occurrence of active disease in SLE.

## INTRODUCTION

Systemic lupus erythematosus (SLE) is a chronic inflammatory disease characterized by abnormal immune responses resulting in the production of autoantibodies, generation of immune complexes, and activation of the complement system. During the course of the disease, patients experience active and/or inactive disease, and multiple organs—including skin, joints, kidney, brain, and serosa—are involved. Corticosteroids and immunosuppressive drugs are used to control the clinical manifestations of SLE, and the prognosis of patients has improved as a result. However, in recent years, atherosclerotic events have been noted among long-surviving patients.^1^

The mechanisms responsible for acceleration of disease activity and promotion of atherosclerosis in SLE are not clearly understood. However, immune responses could be modulated by exposure to different environmental factors such as infectious agents, chemicals, smoking, and diet.^2^^,^^3^ There are reports indicating that dietary factors are related to the risk of atherosclerotic disease among the general population.^4^^,^^5^ Furthermore, studies using mouse models have shown that dietary factors may be associated with the development of autoimmune disease resembling human SLE.^6^^,^^7^ Thus, environmental factors, including lifestyle and diet, may play important roles in the progression of SLE.

Regarding the development of atherosclerosis in SLE, an association with elevated blood homocysteine levels has recently been found.^8^^,^^9^ Vitamins B6 and B12 and folate are known to be important cofactors in the metabolism of homocysteine.^10^ A relationship between dietary fiber intake and homocysteine levels has also been suggested in healthy subjects.^11^ Dietary intakes of vitamin B6 and B12, folate, and dietary fiber, which may determine homocysteine levels, may affect the clinical course of SLE.

To investigate the association of lifestyle and diet with the clinical course of disease in SLE patients, we started the Miyagi Lupus Cohort Study in 1995.^12^ In this cohort study, changes in disease activity and atherosclerotic vascular events were chosen as outcome indicators. The associations between several dietary factors and the clinical course of SLE have been analyzed, and a previous analysis of 4-year follow-up data demonstrated an inverse association between vitamin C intake and the risk of active disease.^13^ In the present analysis, we hypothesized that dietary intakes of vitamins B6 and B12, folate, and dietary fiber might influence the clinical course of women with SLE and investigated the association between intakes of these nutrients and disease activity and atherosclerotic vascular events. Nutrient intakes were estimated using an updated food composition table.^14^ The risk of active disease was evaluated using the 4-year follow-up data. The risk of atherosclerotic vascular events was investigated on the basis of extended follow-up data.

The prevalence of SLE is much higher in women than in men—about 90% of SLE patients are women.^15^ Sex may confound or modify the association between lifestyle and the clinical course of the disease. In the present study, we focused on the clinical course of female patients.

## METHODS

### The Miyagi Lupus Cohort

The study participants were recruited from rheumatology and nephrology departments at 21 hospitals and 2 rheumatology clinics located in Miyagi Prefecture, in northeastern Japan. All the medical institutions are designated SLE referral clinics. Female SLE patients visiting these institutions during the period from 1 June to 30 September 1995 were recruited for the study. During this period, a self-administered questionnaire was handed out to 311 female patients who fulfilled the 1982 revised criteria for the classification of SLE. Of these patients, 279 consented to participate in the study and completed the questionnaires (89.7%).

The patients were also clinically evaluated by their principal physicians. After performing a physical examination, the physicians completed a form that requested information on clinical findings and laboratory data to assess disease activity, organ damage (including atherosclerotic vascular diseases), and medications. The data obtained from the questionnaire survey and the clinical data from the physicians were combined and used as baseline data for the Miyagi Lupus Cohort Study. Details of the study design have been described elsewhere.^12^^,^^13^ This study was approved by the ethical review board of Tohoku University Graduate School of Medicine.

### Baseline questionnaire survey

The questionnaire used in the baseline survey covered general lifestyle factors such as smoking and diet, reproductive history, physical and psychological status, and socioeconomic status, including educational level. Dietary history was assessed using a semiquantitative food frequency questionnaire (FFQ).^16^ Briefly, subjects reported the average frequency of intake of 169 food items and the usual serving size of each item during the year before the baseline survey. Based on the reported frequency of intake and size of each serving, their respective nutrient intakes were computed using the Japanese Standard Tables of Food Composition, fourth and fifth editions.^14^ The FFQ has been validated for the composition tables.^16^^,^^17^ In a comparison of estimates of selected nutrient intake from the questionnaire with estimates from 12 daily diet records kept over a 1-year period, the Spearman correlation coefficients were 0.59, 0.24, 0.46, and 0.60 for vitamins B6 and B12, folate, and dietary fiber, respectively.^17^ Regarding dietary fiber, intakes of soluble and insoluble fiber were also estimated, although, in the fifth edition, the composition data for soluble and insoluble fiber were incomplete for some foods.

### Baseline clinical assessment

Disease activity was evaluated according to the Lupus Activity Criteria Count (LACC); patients with counts of any 2 items or more were classified as having active disease.^18^ At baseline, 34 patients had active disease and 241 had inactive disease. In 4 patients, disease activity was not identified. Although, in clinical trials, several continuous scales are recommended as outcome measures of disease activity, we decided that a dichotomous outcome in the LACC was most appropriate for evaluating associations between dietary factors and disease activity in an observational study. The diagnostic accuracy of LACC was confirmed in our previous report: in the baseline sample of our study, the LACC had 91.2% sensitivity and 77.8% specificity.^13^ These test characteristics are comparable to those reported by Urowitz et al.^18^ Organ damage at baseline, including atherosclerotic vascular diseases, was counted using the Systemic Lupus International Collaborating Clinics/American College of Rheumatology Damage Index (SLICC/ACR DI).^19^ The presence of hypertension and hyperlipidemia was based on physician assessment. Laboratory data in routine examination and information on prednisolone dose were also obtained from physicians.

### Follow-up and identification of active disease and atherosclerotic vascular events

The follow-up survey was conducted in the years 2000 and 2006. In 2000, information on death, relocation, and occurrences of active disease and major organ damage (angina or myocardial infarction, cerebrovascular disease, peripheral vascular disease, end-stage renal disease, aseptic necrosis of the femoral head, and cancer) during 1995–1999 was collected. In the extended survey of 2006, death, relocation, and occurrence of organ damage during 2000–2005 were investigated. All information was obtained from physicians. Details of the survey in 2000 have been described in our previous report.^13^

Occurrence of active disease was evaluated based on the first follow-up survey of 2000. Among the 241 patients who had inactive disease at the baseline, 4 who could not be followed were excluded. Disease activity in 1 patient was not identified in the follow-up survey. Among the remaining 236 patients with inactive disease, 216 with complete dietary data were extracted for analyzing the association of nutrient intake with the risk of active disease (population at risk for active disease).

Atherosclerotic vascular events (occurrence of angina or myocardial infarction, cerebrovascular disease, and peripheral vascular disease) were identified based on both the follow-up survey in 2000 and the extended survey in 2006. In the cross-sectional analysis of baseline data, we found some differences in nutrient intake between patients who had active and inactive disease: patients with active disease consumed a smaller amount of total fat and a larger amount of carbohydrate than those in the inactive phase (data not shown). It is likely that disease activity at baseline confounded the association between dietary factors and the risk of atherosclerotic vascular events. Thus, the 215 patients with no history of vascular events who had inactive disease at baseline were regarded as eligible. Among them, the 196 patients with complete dietary data were selected for evaluating the risk of atherosclerotic vascular events (population at risk for atherosclerotic vascular events).

### Statistical analysis

Length of follow-up for each patient was calculated from the date of the baseline survey to the date of diagnosis of active disease or atherosclerotic vascular event, the date of death, the date of relocation or loss to follow-up, or the date the follow-up survey was closed. In the analyses, we categorized patients who developed angina or myocardial infarction, cerebrovascular disease, and peripheral vascular disease into 1 group (patients with atherosclerotic vascular events).

The associations of nutrient intake with the risk of active disease and the risk of atherosclerotic vascular events were estimated using Cox proportional hazards models.^20^ Nutrient intake at baseline was adjusted for energy intake and categorized by tertiles based on the distribution among the population at risk for each outcome.^21^ Hazard ratios (HRs) and 95% confidence intervals (95% CIs) for each category of intake were computed, using the lowest tertile as reference category. Tests of trend across categories of nutrient intake were conducted in the models by treating the median values of the categories as continuous variables. Adjustments were also made for nondietary factors, which included age, duration of disease, habitual smoking, education level, prednisolone dose, body mass index (BMI), and SLICC/ACR DI score. Additionally, pregnancy after disease onset was adjusted for in the analysis of active disease risk because it has been identified as a risk factor for disease flare.^22^ In the analysis of atherosclerotic vascular events, traditional risk factors for atherosclerotic disease, eg, hypertension and hyperlipidemia, were also controlled for.^23^^,^^24^

The questionnaire used in this study included items on vitamin supplement use; however, intakes via supplements were not included in the computation of nutrient intakes because no comprehensive data on the constituents of the supplements were available. Alternatively, we performed additional analyses after exclusion of subjects who had used vitamin supplements.

Results were regarded as significant if 2-sided *P* values were less than 0.05. All statistical analyses were performed using the SAS software package (version 9.1; SAS Institute, Cary, NC, USA).

## RESULTS

The baseline characteristics of the 2 study populations (populations at risk for active disease and atherosclerotic vascular events) are shown in Table 1. Both populations had similar mean disease duration and mean age. Most subjects were taking steroids.

**Table 1.** Baseline characteristics of population at risk in the Miyagi Lupus Cohort Study

	Population at risk for active disease (*n* = 216)		Population at risk for atherosclerotic vascular events (*n* = 196)	
		
	Mean^a^	Number (%)	Mean^a^	Number (%)
Age (years)	40.6 ± 13.3		40.0 ± 13.5	
Duration of disease (years)	10.1 ± 6.9		9.8 ± 7.0	
Habitual smoking		26 (12.0)		24 (12.2)
Education (junior high school or lower)		33 (15.3)		30 (15.3)
Pregnancy after disease onset		43 (19.9)		38 (19.4)
Body mass index (kg/m^2^)				
<25		186 (86.1)		167 (85.2)
25≤		30 (13.9)		29 (14.8)
Prednisolone dose (mg)^b^				
0		11 (5.1)		11 (5.6)
≤10		159 (73.6)		145 (74.0)
10< to <20		41 (19.0)		35 (17.9)
20≤		5 (2.3)		5 (2.6)
SLICC/ACR DI score	1.1 ± 1.1		1.0 ± 1.0	
Physical status				
Diabetes mellitus		12 (5.6)		11 (5.6)
Hyperlipidemia		26 (12.0)		25 (12.8)
Hypertension		45 (20.8)		36 (18.4)
Renal dysfunction (serum creatinine ≥1.0 mg/dl)		43 (19.9)		20 (10.2)
Cerebrovascular disease		3 (1.4)		0 (0.0)
Angina or myocardial infarction		11 (5.1)		0 (0.0)
Peripheral vascular disease		9 (4.2)		0 (0.0)
Nutrient intake/day				
Total energy (Kcal)	2316.4 ± 1023.2		2318.7 ± 1034.3	
Total protein (g)	87.5 ± 11.2		87.8 ± 11.3	
Carbohydrate (g)	300.9 ± 27.9		300.0 ± 28.3	
Total fat (g)	61.6 ± 8.6		61.8 ± 8.7	
Vitamin C (mg)	176.2 ± 73.3		171.1 ± 67.1	
Vitamin D (µg)	8.3 ± 4.3		8.2 ± 4.4	
Vitamin E (mg)	10.7 ± 1.9		10.7 ± 1.8	

Abbreviation: SLICC/ACR DI, Systemic Lupus International Collaborating Clinics/American College of Rheumatology Damage Index.

^a^Mean ± SD (all such values).

^b^Dose of steroid prescribed was converted to equivalent prednisolone dose.

### Risk of active disease

Among the 216 subjects at risk, 4 (1.8%) relocated during the period 1995–1999. Consequently, after an average 46.1-month follow-up, corresponding to 9966 person-months, 43 patients developed active disease. The serological profiles of these 43 patients were described in our previous report.^13^

Table 2 shows the associations of the nondietary factors listed in Table 1 with the risk of active disease. These nondietary factors were taken into account in the analyses examining the associations between dietary intake and the risk of active disease. Pregnancy was not related to the risk of active disease. Higher BMI tended to be associated with a lower risk of active disease.

**Table 2.** Hazard ratios (HRs) and 95% confidence intervals (CIs) associated with risk of active disease according to nondietary factors

Factor	HR^a^	95% CI
Age (years)	0.98^b^	0.96–1.01
Duration of disease (years)	0.99	0.94–1.04
Habitual smoking	0.72	0.26–2.01
Lower education level^c^ (junior high school or lower)	1.20	0.48–3.00
Pregnancy after disease onset	0.93	0.43–2.02
Body mass index 25≤^d^	0.29	0.07–1.22
Dose of prednisolone^e^	1.44	0.88–2.34
SLICC/ACR DI score^f^	1.11	0.86–1.43

Abbreviation: SLICC/ACR DI, Systemic Lupus International Collaborating Clinics/American College of Rheumatology Damage Index.

^a^Cox proportional hazards model was used to estimate HRs and 95% CIs; HRs were adjusted for age.

^b^HR estimated for age only.

^c^Education level of high school or higher was defined as the referent category.

^d^Body mass index <25 was defined as the referent category.

^e^Prednisolone dose was categorized into the 4 dose levels shown in Table 1. Dose level was treated as a continuous variable.

^f^SLICC/ACR DI score was treated as a continuous variable.

Table 3 shows the associations between nutrient intake and the risk of active disease. It includes age- and disease duration-adjusted HRs as well as HRs estimated in the multivariate models that included nondietary factors. The multivariable-adjusted HRs were similar to the age- and disease duration-adjusted HRs. In the multivariate model, a significant inverse association was observed between vitamin B6 intake and the risk of active disease (*P* for trend = 0.04): the HR for the highest vs lowest tertile of vitamin B6 intake was 0.41 (95% CI: 0.18–0.97), indicating that higher vitamin B6 intake was associated with a reduced risk of active disease independently of nondietary factors. A similar inverse association was observed for folate intake; however, this association was nonsignificant. Intake of total dietary fiber was inversely associated with the risk of active disease (*P* for trend = 0.01): the HR for the highest vs lowest tertile of the intake of total dietary fiber was 0.29 (95% CI: 0.11–0.78). The analysis of soluble and insoluble fiber also showed inverse associations: the HR for the highest vs lowest tertile was 0.43 (95% CI: 0.18–0.99) for soluble fiber and 0.39 (95% CI: 0.15–0.97) for insoluble fiber. There was no association between vitamin B12 intake and the risk of active disease.

**Table 3.** Hazard ratios (HRs) and 95% confidence intervals (CIs) associated with risk of active disease according to tertiles of energy-adjusted daily nutrient intake^a^

	Low	Middle	High	*P* for trend
Vitamin B6 (mg)	<1.5	1.5–1.7	1.7≤	
No. of cases/person-months	20/3161	14/3263	9/3542	
Age- and disease duration-adjusted HR (95% CI)^b^	1.00	0.72 (0.36–1.46)	0.43 (0.19–0.98)	0.04
Multivariable-adjusted HR (95% CI)^c^	1.00	0.73 (0.35–1.50)	0.41 (0.18–0.97)	0.04

Vitamin B12 (µg)	<7.5	7.5–10.2	10.2≤	
No. of cases/person-months	13/3403	17/3200	13/3363	
Age- and disease duration-adjusted HR (95% CI)	1.00	1.30 (0.63–2.68)	1.04 (0.48–2.27)	0.98
Multivariable-adjusted HR (95% CI)	1.00	1.21 (0.58–2.52)	1.06 (0.49–2.33)	0.92

Folate (µg)	<404.8	404.8–523.0	523.0≤	
No. of cases/person-months	18/3186	15/3296	10/3484	
Age- and disease duration-adjusted HR (95% CI)	1.00	0.85 (0.43–1.71)	0.56 (0.25–1.28)	0.17
Multivariable-adjusted HR (95% CI)	1.00	0.93 (0.45–1.90)	0.58 (0.25–1.33)	0.19

Total dietary fiber (g)	<15.0	15.0–20.0	20.0≤	
No. of cases/person-months	20/3148	17/3307	6/3511	
Age- and disease duration-adjusted HR (95% CI)	1.00	0.82 (0.42–1.60)	0.28 (0.11–0.74)	0.01
Multivariable-adjusted HR (95% CI)	1.00	0.86 (0.44–1.71)	0.29 (0.11–0.78)	0.01

Soluble dietary fiber (g)	<3.6	3.6–4.7	4.7≤	
No. of cases/person-months	21/3109	13/3301	9/3556	
Age- and disease duration-adjusted HR (95% CI)	1.00	0.61 (0.30–1.23)	0.40 (0.18–0.92)	0.03
Multivariable-adjusted HR (95% CI)	1.00	0.67 (0.33–1.36)	0.43 (0.18–0.99)	0.04

Insoluble dietary fiber (g)	<10.9	10.9–14.1	14.1≤	
No. of cases/person-months	19/3165	17/3224	7/3577	
Age- and disease duration-adjusted HR (95% CI)	1.00	0.95 (0.48–1.88)	0.36 (0.15–0.89)	0.02
Multivariable-adjusted HR (95% CI)	1.00	0.98 (0.49–1.96)	0.39 (0.15–0.97)	0.04

^a^Cox proportional hazards model was used to estimate HRs and 95% CIs.

^b^Adjusted for age, duration of disease, and energy intake.

^c^Adjusted for age, duration of disease, habitual smoking, education level, pregnancy after disease onset, body mass index, prescribed prednisolone dose, Systemic Lupus International Collaborating Clinics/American College of Rheumatology Damage Index score, and energy intake.

A total of 27 patients reported taking vitamin supplements at least once a week at baseline. Exclusion of these patients somewhat strengthened the inverse associations for the intake of vitamin B6 and dietary fiber: the HR for the highest vs lowest tertile of vitamin B6 intake was 0.38 (*P* for trend = 0.04), and that for dietary fiber intake was 0.21 (*P* for trend = 0.008) (data not shown in tables).

### Risk of atherosclerotic vascular events

Of the 196 subjects at risk, 4 relocated during the period of the first survey (1995–1999). During the period of the extended survey (2000–2005), 21 subjects relocated and 14 were lost to follow-up. Finally, after an average 99.9-month follow-up for the 196 subjects at risk, corresponding to 19 575 person-months, 20 patients developed atherosclerotic vascular events (angina or myocardial infarction, 8; cerebrovascular disease, 7; peripheral vascular disease, 5).

Table 4 shows HRs of known risk factors for atherosclerotic diseases and other nondietary factors. The presence of diabetes mellitus and renal dysfunction at baseline tended to be associated with an increased risk of atherosclerotic vascular events, whereas the association with the presence of hypertension and hyperlipidemia and smoking was unity. Patients with lower education levels had a higher risk of atherosclerotic vascular events: the HR for lower education (junior high school or lower) as compared with higher education (high school or higher) was 3.56 (95% CI: 1.30–9.73). The HR for higher BMI was slightly elevated. Prednisolone dose was positively associated with the risk of atherosclerotic vascular events; however, statistical testing showed only borderline significance.

**Table 4.** Hazard ratios (HRs) and 95% confidence intervals (CIs) associated with risk of atherosclerotic vascular events according to nondietary factors

Factor	HR^a^	95% CI
Age (years)	1.05^b^	1.01–1.09
Duration of disease (years)	1.01	0.96–1.07
Habitual smoking	1.18	0.27–5.23
Lower education level (junior high school or lower)^c^	3.56	1.30–9.73
Body mass index 25≤^d^	1.35	0.48–3.76
Diabetes mellitus	2.75	0.63–12.00
Hyperlipidemia	1.27	0.41–3.90
Hypertension	1.04	0.37–2.94
Renal dysfunction (Serum creatinine ≥1.0 mg/dl)^e^	3.25	0.93–11.34
Prednisolone dose^f^	2.04	0.97–4.29

^a^Cox proportional hazards model was used to estimate HRs and 95% CIs; HRs were adjusted for age.

^b^HR estimated for age only.

^c^Education level of high school or higher was defined as the referent category.

^d^Body mass index <25 was defined as the referent category.

^e^Serum creatinine <1.0 mg/dl was defined as the referent category.

^f^Prednisolone dose was categorized into the 4 dose levels shown in Table 1. Dose level was treated as a continuous variable.

Table 5 shows the associations between nutrient intakes and the risk of atherosclerotic vascular events. In the age- and disease duration-adjusted model, vitamin B6 intake was inversely associated with the risk of atherosclerotic vascular events: the HR for the highest vs lowest tertile of intake of vitamin B6 was 0.43 (95% CI: 0.19–0.98). However, this association became insignificant after further adjustment for nondietary factors, including known risk factors of atherosclerotic disease. Intake of insoluble dietary fiber tended to be inversely associated with the risk of atherosclerotic vascular events in both the age- and disease duration-adjusted and multivariate models; however, the test for trend showed that the association was nonsignificant (*P* for trend in the multivariate model = 0.13).

**Table 5.** Hazard ratios (HRs) and 95% confidence intervals (CIs) associated with risk of atherosclerotic vascular events according to tertiles of energy-adjusted daily nutrient intake^a^

	Low	Middle	High	*P* for trend
Vitamin B6 (mg)	<1.5	1.5–1.7	1.7≤	
No. of cases/person-months	6/6380	9/6699	5/6496	
Age- and disease duration-adjusted HR (95% CI)^b^	1.00	1.03 (0.35–3.05)	0.43 (0.19–0.98)	0.16
Multivariable-adjusted HR (95% CI)^c^	1.00	1.04 (0.35–3.10)	0.41 (0.10–1.72)	0.22

Vitamin B12 (µg)	<7.6	7.6–10.2	10.2≤	
No. of cases/person-months	5/6339	4/6481	11/6755	
Age- and disease duration-adjusted HR (95% CI)	1.00	0.83 (0.22–3.09)	1.72 (0.59–5.03)	0.24
Multivariable-adjusted HR (95% CI)	1.00	0.87 (0.23–3.35)	1.86 (0.60–5.82)	0.22

Folate (µg)	<404.4	404.4–520.9	520.9≤	
No. of cases/person-months	6/6589	5/6580	9/6406	
Age- and disease duration-adjusted HR (95% CI)	1.00	0.64 (0.19–2.18)	0.84 (0.25–2.81)	0.87
Multivariable-adjusted HR (95% CI)	1.00	0.56 (0.16–1.99)	0.83 (0.23–2.99)	0.91

Total dietary fiber (g)	<14.9	14.9–19.9	19.9≤	
No. of cases/person-months	4/6758	9/6159	7/6658	
Age- and disease duration-adjusted HR (95% CI)	1.00	1.80 (0.53–6.16)	0.96 (0.25–3.73)	0.74
Multivariable-adjusted HR (95% CI)	1.00	1.69 (0.48–6.02)	0.89 (0.21–3.74)	0.68

Soluble dietary fiber (g)	<3.6	3.6–4.7	4.7≤	
No. of cases/person-months	5/6671	8/6287	7/6617	
Age- and disease duration-adjusted HR (95% CI)	1.00	1.25 (0.40–3.94)	0.79 (0.23–2.76)	0.63
Multivariable-adjusted HR (95% CI)	1.00	1.61 (0.46–5.66)	0.83 (0.22–3.15)	0.63

Insoluble dietary fiber (g)	<10.9	10.9–14.1	14.1≤	
No. of cases/person-months	6/6793	9/6094	5/6688	
Age- and disease duration-adjusted HR (95% CI)	1.00	0.98 (0.32–2.99)	0.44 (0.12–1.58)	0.16
Multivariable-adjusted HR (95% CI)	1.00	0.90 (0.28–2.91)	0.39 (0.10–1.51)	0.13

^a^Cox proportional hazards model was used to estimate HRs and 95% CIs.

^b^Adjusted for age, duration of disease, and energy intake.

^c^Adjusted for age, duration of disease, habitual smoking, education level, body mass index, number of preexisting diseases (diabetes mellitus, hyperlipidemia, hypertension, and renal dysfunction), prescribed prednisolone dose, and energy intake.

After excluding the 21 users of vitamin supplements, we reevaluated the association for nutrient intake; the results were essentially unchanged (data not shown in tables).

## DISCUSSION

In the present prospective study of patients with SLE, we found that intakes of vitamin B6 and dietary fiber were inversely associated with the risk of active disease. There was no significant association between intake of these nutrients and the risk of atherosclerotic vascular events. These results remained essentially unchanged after exclusion of vitamin supplement users.

Before interpreting our results, some methodological problems merit consideration. The present study had a number of strengths and limitations. Strengths included its prospective design and consideration of confounding by clinical and other nondietary factors. Dietary habits among subjects suffering from chronic diseases such as SLE can be influenced by their symptoms, physical condition, and medication. Thus, the prospective design and adjustments for clinical factors were essential for clarifying the cause-effect relationship between nutrient intake and the clinical course of disease. The limitations were as follows. First, the possibility of selection bias in recruiting the study subjects must be considered. However, the subjects were recruited from referral clinics for SLE. The response rate in the questionnaire survey was relatively high, although several seriously ill inpatients might not have participated in the study. We therefore believe that our results represent the characteristics of female SLE patients residing in the study area. Second, loss to follow-up might have influenced the results. The follow-up among the population at risk for active disease was approximately complete. However, of 196 subjects at risk of atherosclerotic vascular events, 39 were not completely followed up because of relocation or loss to follow-up; the mean follow-up period in these 39 subjects was 58.4 months. In particular, the rate of relocation and loss to follow-up in subjects younger than 30 years was high, which might have distorted the results. Thus, after excluding these younger subjects, we reanalyzed the data. However, the results of this reanalysis were similar to the present findings. Therefore, we believe that relocation and loss to follow-up did not have a significant effect on the results. Third, the problem of limited statistical power must be considered; the small sample size and the relatively small number of atherosclerotic vascular events might have been problematic. Also, differences in diagnostic ability among the physicians might have affected the number of events reported by each hospital. However, it is unlikely that the incidence of events would have been under- or overestimated in our study.^25^^,^^26^ We believe that our findings warrant discussion, although careful interpretation is necessary. Fourth, we were unable to collect blood samples from the study subjects, and thus serum levels of vitamins, folate, homocysteine, and specific inflammatory markers were not measured. The biological plausibility of our results was discussed on the basis of nutrient intake estimated from the questionnaire data.

With regard to micronutrients analyzed in the present study, several health effects are known. Intakes of vitamin B6 and B12, folate, and dietary fiber may influence serum levels of inflammation markers such as C-reactive protein (CRP), cytokines, and homocysteine.^11^^,^^27^^–^^29^ Moreover, higher intake of these nutrients may decrease the risk of atherosclerotic diseases in the general population by controlling serum levels of some cytokines and homocysteine.^30^^–^^32^ In SLE, the association of a higher serum level of homocysteine with atherosclerotic vascular events has been indicated, and vitamin interventions for atherosclerotic vascular events have been proposed.^8^^,^^9^ However, there has been no investigation of whether intake of vitamins and dietary fiber modifies the clinical course of SLE, including disease activity and atherosclerotic vascular events. Therefore, no prior evidence is available for SLE. Nevertheless, there is biological plausibility for the significant findings in this study, namely, the inverse associations of vitamin B6 and dietary fiber intakes with the risk of active disease. First, it is possible that a higher vitamin B6 intake might have decreased the risk of active disease by decreasing the level of homocysteine. An inverse association between intakes of vitamin B6 and folate and serum homocysteine concentration has been observed in Japanese women.^17^ Several previous studies have indicated that hyperhomocysteinemia may be related not only to the development of atherosclerosis, but also active inflammation and immune activation in autoimmune diseases such as rheumatoid arthritis.^33^^–^^36^ Hyperhomocysteinemia may be related to an increased level of some inflammatory markers,^37^ which may contribute to the progression of active disease.^38^^,^^39^ Brown found that monocyte chemoattractant protein-1 concentrations in plasma were strongly positively correlated with homocysteine concentrations in patients with SLE, indicating that active inflammation in SLE and hyperhomocysteinemia may be etiologically linked.^40^ Higher intake of vitamin B6 may prevent hyperhomocysteinemia, which may lead to the suppression of active inflammation, ie, active disease. Second, there is a possibility that vitamin B6 status may directly modulate disease activity, independently of homocysteine levels. In the metabolism of antibodies and cytokines, vitamin B6 and folate act as coenzymes.^10^^,^^27^ Deficiency of vitamin B6 and folate impairs lymphocyte maturation.^27^^,^^41^^,^^42^ Thus, a higher intake of vitamin B6 may improve overall immune function and suppress disease activity.

The association of dietary fiber intake with disease activity may be due to mechanisms similar to those mentioned above for intake of vitamin B6, although specific effects according to the type of dietary fiber cannot be defined. Some previous studies have shown that dietary fiber intake is inversely associated with the plasma level of homocysteine and inflammatory markers including interleukin-6 and CRP.^11^^,^^32^^,^^43^ Higher intake of dietary fiber might decrease the risk of active disease by decreasing the levels of some cytokines and homocysteine. In addition, dietary fiber has a unique effect that differs from that of vitamin B6. Higher intake of dietary fiber promotes gut mobility and lessens the time available for absorption of some harmful compounds from the gut and thus may contribute to the reduction in serum levels of harmful substances such as phenols.^44^ It has been hypothesized that increased levels of these substances are related to rheumatoid symptoms.^45^^,^^46^ The inverse association between dietary fiber intake and disease activity may be partly explained by this intriguing effect of dietary fiber.

We found no statistically significant association between the intake of nutrients investigated in the present study and the risk of atherosclerotic vascular events. In SLE, some factors, including traditional Framingham risk factors, have already been established as risk factors of atherosclerotic events.^47^ We have considered the possibility that similarities in associations with nutrient intake might exist between atherosclerotic disease in the general population and patients with SLE. However, associations with intake of vitamin B6 and folate described in the general population were not observed in the present study.^48^ This nonsignificant finding suggests that complex mechanisms may underlie the effects of dietary factors on the progression of atherosclerosis in SLE, despite the possibility of limited statistical power in the analysis. Recent studies have shown that the occurrence of atherosclerotic disease in SLE might be mediated not only by traditional risk factors but also disease-related factors such as immune complex formation.^49^^,^^50^ Thus, the significant associations of vitamin B6 and dietary fiber intakes with disease activity mentioned above may indicate that nutrient intake modifies the risk of atherosclerotic vascular events by mediating disease activity. In future studies, it will be necessary to investigate the interrelations among dietary factors, disease activity, and the progression of atherosclerosis.

In conclusion, the present study revealed associations of selected vitamins, including vitamins B6, B12, and folate, and dietary fiber with clinical disease course in women with SLE. Intake of vitamin B6 and dietary fiber was inversely associated with the risk of active disease. There was no significant association between intake of these nutrients and the risk of atherosclerotic vascular events. These findings suggest that intake of vitamin B6 and dietary fiber may prevent the occurrence of active disease in SLE patients. In subsequent studies, the associations between intake of these nutrients and serum levels of inflammatory markers in SLE patients will need to be evaluated. For generalization of our results, especially the result on atherosclerotic vascular events, large-scale studies will also be required.
